# Dark secrets behind the shimmer of contact lens: the Indian scenario

**DOI:** 10.1186/1756-0500-2-79

**Published:** 2009-05-09

**Authors:** Lekha Tuli, Gopal Krishna Bhatt, Deepak Kumar Singh, Tribhuban Mohan Mohapatra

**Affiliations:** 1 Department of Microbiology, Institute of Medical Sciences, Banaras Hindu University, Varanasi, India; 2 Institute of Medical Sciences, Banaras Hindu University, Varanasi, India; 3 Department of Microbiology, Kasturba Medical College, Manipal University, Mangalore, India

## Abstract

**Background:**

We studied the bacteriological profile of soft contact lens and its accessories among the asymptomatic subjects and monitored the compliance level with the lens use and its cleaning protocol.

**Findings:**

A total of 115 (104 daily wear and 11 extended wear) subjects using contact lens were studied. Data regarding the duration of use and frequency and method of cleaning were recorded. Contact lens, lens cases, preserving solutions and tips of solution bottles were the samples collected. The isolates were identified on the basis of their phenotypic characters. Samples from 24 subjects (21 daily wear and 3 extended wear) were found contaminated. Of the 24 contaminated cases, 23 showed medium adherence to the cleaning protocol. Contamination rate was higher among the 56 daily wear lens users who used same lens for 2 years and more, than the 48 users who used their lenses for less than 2 years. Lens case contamination was found in all the 24 cases. The bacteria isolated were *Citrobacter freundii, Escherichia coli*, *Klebsiella pneumoniae, Pseudomonas aeruginosa*, *Staphylococcus epidermidis *and *Streptococcus pneumoniae*. In extended wear lens users, there was no change in microbial flora on repeating the cultures on day 7 and 14.

**Conclusion:**

Non-compliance with contact lens use may lead to invitation of microbial flora. The accumulation of these bacteria may act as a precursor to biofilm formation, thus colonizing the lens accessories as well. The bacteria isolated in this study were similar to the ones causing microbial keratitis thus, predisposing the otherwise asymptomatic subjects to permanent visual damage.

## Background

Contact lenses have been used as refractive aid from the Leonardo de Vinci days (1508). Since then, the quality of lens and their uses has undergone a metamorphosis. Besides having optical, protective and therapeutic significance, contact lenses are widely used as cosmetic aids by the newer generation [1]. However, the lens can alter the normal physiology of the cornea by causing hypoxia, alterations in tear film dynamics and mechanical trauma. Even mild micro trauma may lead to microbial invitation [2]. Contamination of the contact lens cases, solution, lens material, wearing schedule and disinfection techniques are the important factors that influence infections related to contact lens use.

Contact lens when inserted into the eye, rapidly accumulate proteins, glycoproteins and lipids from tear film on its surface providing a conducive environment for bacterial adhesion. The ability of adhered bacteria to grow on the tear film components adsorbed on the lens surface is a pathogenic trait. They proliferate forming micro colonies that coalesce to form biofilms. There have been reports of isolating identical microorganisms from contact lens case and that of keratitis [3]. Studies reveal that bacterial adhesion to contact lenses is clearly involved in production of several adverse responses [4].

Non-compliance with contact lens care and hygiene may result in their contamination predisposing the eye to infections. Microbial keratitis is one of the serious complications of contact lens use and if not treated timely may result in permanent visual damage to the cornea [5].

The objective of the present study was to determine the bacteriological profile of the soft contact lens and its accessories during use and also to look at the role of regular cleaning and disinfection procedures in minimizing the risk of contact lens contamination and biofilm formation.

## Methods

### Study cases

This study was conducted on 115 college going students who used contact lens regularly. The samples were collected after duly informing them that they were participating in a research study. For all assignments of this type institute ethical committee first review the protocol then approves it. Most of the subjects were asymptomatic except 4 cases that presented with mild irritation in the eyes during use of contact lens but they did not have any problems with the regular lens use.

A questionnaire was prepared and data regarding the age, sex, type of lens, duration of use, method of cleaning and frequency of changing the accessories was recorded.

In case of daily wear use, the user removed the lens each night, cleaned and immersed them overnight in lens solution, returning the same lens to the eyes the next day. Whereas, in extended wear lens, the user wore the same lens continuously for, commonly 6 nights and replaced it with a new one on the seventh day [4].

High adherence to compliance indicated washing of hands before lens removal, immersing the lens in clean solution overnight and discarding the overnight solution before reimmersing the lens. Medium adherence to compliance indicated violation of any one of the above steps. Low adherence to compliance indicated two or more violations of the above steps.

### Laboratory procedures

A total of 115 cases using extended wear and daily wear lenses were studied. From 11 students who used extended wear lens, a repeat of samples on day 7 and day 14 was asked for. Samples from these users were taken on the 7th day before discarding. These were cultured to check for any growth. The procedure was repeated for the new lens, which was used for a week i.e. the subjects were called on the 14th day in order to find out as to which of the four things (lens/case/solution/bottle tip) was actually contaminated. In case of daily wear lens overnight samples were collected.

The cleaning solutions for the contact lenses were cultured with the sterile bacteriologic loop. Rayon swabs were used to take samples from the lens and from the concave surface of the lens containers after discarding the solution. The tips of the solution bottles were pressed directly onto the surface of the media.

Samples were inoculated into tryptic soy broth, blood agar, Maconkey's agar and Sabouraud's dextrose agar, incubated at 37°C for 24 to 48 hrs and examined for bacterial growth. Sabouraud's dextrose agar was incubated at 25°C, examined daily and discarded at the end of 3 weeks if there was no growth.

Microbial cultures were considered significant if there was confluent growth at inoculation site, if the growth on the medium coincided with microscopic findings (i.e. Gram stain) and if the same organism grew on more than one media [3]. Gram stain was performed for all the isolates recovered. The isolates were identified on the basis of their phenotypic characters following standard laboratory protocols.

## Results

Of the 115 cases studied, there were 89 (77%) females and 26 (22%) males, all in the age group of 18–25 years (Table 1).

**Table 1:** Summary of the Questionnaire Responses from the subjects (n = 115)

	Extended Wear Lens	Daily Wear Lens
**1. Sex**		
a. Female	9(7.8%)	80(69.56%)
b. Male	2(1.73%)	24(20.86%)

**2. Duration of use**		
≥ 2 years: contaminated cases	-	56(48.69%):16
< 2 years: contaminated cases	-	48(41.73%):5

3. Cleaning Schedule*		
(a)High Adherence: contaminated cases	7(6.09%): 0	78(67.82%): 1

(b)Medium Adherence: contaminated cases	4(3.48%): 3	26(22.60%): 20

(c) Low adherence: contaminated cases	0:0	0:0

**4. Frequency of changing accessories.**		

Before 1 year: contaminated cases	9(7.82%):1	91(79.13%):16

After 1 year: contaminated cases	2 (1.73%):2	13(11.3%):5

**5. Reasons for use**		

Cosmetic	2(1.73%)	4(3.47%)

Therapeutic	2(1.72%)	1(0.86%)

Cosmetic and Therapeutic	7(6.08%)	99(86.08%)

*Cleaning schedule included the steps followed for cleaning the lens.

High adherence to compliance indicated washing of hands before lens removal, immersing in clean solution overnight and discarding the overnight solution before re immersing the lens.

Medium adherence to compliance indicated violation of any one of the above steps.

Low adherence to compliance indicated two or more violations of the above steps.

In case of extended wear use, the user wears the same lens continuously for, commonly 6 nights, then removes the lens and inserts a new lens on the seventh day. The cleaning protocol was the same as for daily wear lens.

Daily wear lens were used by 104 (90.43%) subjects. While interviewing the candidates it was found that 78 (67.82%) followed the above cleaning protocol very strictly (high adherence to compliance). Whereas, 26 (22.60%) showed breaches in following any one of the instructions and were classified as those showing medium adherence to compliance (Table 1). On statistical analysis a significant difference was found in the levels of contamination among the above two groups (Fisher's Exact Test, p < 0.001).

Among the daily wear lens users, in 56 (48.69%) subjects who used same lens for 2 years and more, contamination rate was higher compared to 48 (41.73%) subjects who used their lenses for less than 2 years (Fisher's Exact Test, p = 0.0275). (Table 1). The remaining 11 (9.56%) subjects used extended wear lens.

Samples of 24 (20.86%) cases were contaminated. The remaining 91 samples had no growth. The lens case was the most frequently contaminated item found in all the 24 cases. On comparison with the next most frequently contaminated item i.e. cleaning solution, a significant difference was found between the two (Fishers Exact Test, p = 0.011). Of the 104 cases using daily wear lens 21, and of the 11 students using extended wear lens, 3 were contaminated. In 18 out of 24 (75%), the same organism being isolated from the lens, cleaning solution and lens case. In the remaining 6 cases (25%) cultures from the tip of the solution bottle and lens case were found to have identical organisms. Out of total, three samples showed mixed growth. On repeating the samples on day 7 and 14 after changing the lens, in case of extended wear lens users no change was found in the microbial flora.

The bacteria isolated from the daily wear lens users were *Pseudomonas aeruginosa *(37.5%), *Klebsiella pneumoniae *(24%), *Escherichia coli *(16.67%), *Citrobacter freundii *(8.33%), *Staphylococcus epidermidis *(4.17%) and *Streptococcus pneumoniae *(4.17%). However, among the extended wear lens users the bacteria isolated were *Pseudomonas aeruginosa, Klebsiella pneumoniae *and *Staphylococcus epidermidis *(4.17% each) (Table 2).

**Table 2:** Bacterial isolates from contact lens and accessories

Lab nos. of subjects	Extended wear lens		Daily wear lens	Lens case	Cleaning solutions	Solution bottle tips
2	Ps		-	Ps	Ps	-

8	-		K	K	K	-

10	-		E	E, Ps	E	-

14	-		-	Ps	-	Ps

18	-		-	C	-	C

19	-		E	E	E	-

20	-		K	K	K	-

23	-		K	K	K	-

30	-		-	K	-	K

32	-		SP	SP, E	SP	-

48	-		Ps	Ps	Ps	-

49	-		Ps	Ps	Ps	-

56	-		-	Ps	-	Ps

58	-		E	E	E	-

61	-		SE	SE	SE, C	-

66	-		K	K	K	-

78	SE		-	SE	SE	-

83	-		Ps	Ps	Ps	-

91	-		Ps	Ps	Ps	-

97	-		-	K	-	K

101	-		E	E	E	-

106	-		Ps	Ps	Ps	-

112	K		-	K	K	-

114	-		-	Ps	-	Ps

C – *Citrobacter freundii*

E – *Escherichia coli*

K * – Klebsiella pneumoniae*

Ps – *Pseudomonas aeruginosa*

SE – *Staphylococcus epidermidis*

SP – *Streptococcus pneumoniae*

None of the samples were contaminated with fungus.

## Discussion

Over a decade, contact lens use has gained importance due to cosmetic reasons. With the evolution of time there have been developments from rigid lenses to more than sixty materials being used for manufacturing soft lens [1].

Such a sensitive eye care product needs to be handled carefully. The present study reveals that 25% cases showed medium adherence to compliance to lens use. Collins et al also documented non-compliance of 70% among their subjects [6]. Similarly, Yung et al reported level of non-compliance to be 60% in their study [7]. However, a study from Wills Eye Hospital conducted by Najjar et al showed that 30% cases developed corneal ulcers despite being compliant with the guidelines for contact lens wear [8]. Reports of poor patient compliance in contact lens wear date back to the mid 1980's where patients under 30 years and over 50 years of age were more likely to have poor compliance [9]. There may be a possibility of elderly subjects showing greater lens contamination owing to their poor compliance.

The lens case, being static with relatively low nutrients, provides a favorable environment for biofilm formation. Its manufacturing design, especially the corners, makes it difficult to clean and vulnerable to bacterial colonization. In this study the lens case was observed to be the most frequently contaminated item. Boost et al, in their study, also reported 39% of lens case contamination [10]. Thus, the lens and its accessories need to be changed at regular intervals as users tend to become careless in lens handling inviting bacterial contamination. A significant difference was observed in contamination levels of lens accessories between the groups who changed their lens accessories within a year to those who did not (Fisher's Exact Test, p = 0.0152).

Such high contamination levels in our study could be attributed not only to non-compliance and unhygienic practices by the subjects but also due to lack of communication between them and the practitioners. Shelf buying of the lenses from local lens dealers, a common practice in our country adds to the problem, as they are unable to give proper instructions to the users. Steinemann et al also highlight the problems faced by people due to purchase of contact lens from unlicensed vendors [11].

On applying Fishers Exact test, no significant difference was detected in the incidence of *Pseudomonas aeruginosa *(p = 1); *Klebsiella pneumoniae *(p = 1) and *Staphylococcus epidermidis *(p = 0.239) among daily wear and extended wear lens users. The other three isolates were found only in daily wear lens users. It clearly indicated that use of extended wear lens neither resulted in an increase in the microbial contamination nor in the types of organisms isolated as compared to daily wear lens. Willcox et al in their study found similar results [4]. Thus, contamination of lenses is sporadic. Goodlawsuggested that as the lens are not changed during night in case of extended wear lens, the microbial colonization of the eye can be due to organisms acquired during their use in the daytime [12]. The same organisms as that found on the lens cases colonized the lenses. The most common ocular pathogen observed in this study was *Pseudomonas aeruginosa *(41.66%), which is frequently found in the bathroom environment. Presence of *Klebsiella *spp. and *Escherichia coli *indicated fecal contamination either from unwashed hands, poor hygienic habits or aerosols/surface contamination of the lens accessories stored in the bathroom. Boost et al reported *Acinetobacter spp*. as the most frequently isolated organism followed by *Enterobacter spp*. [10]. Pinna et al reported the first case of contact lens related *Bacillus cereus *keratitis and ulcer associated with *Bacillus cereus *contamination of contact lens case [13]. These isolates did not corroborate with ours. However, in a study conducted by Bharathi et al on cases with infective keratitis, similar organisms have been isolated as in the present study, their isolates being *Pseudomonas *spp., *Staphylococcus epidermidis *and *Streptococcus pneumonia *[3].

In India, most of the contact lens cleaning solutions either contain tauranol or poloxamer which when used alone might not be sufficient enough for debris removal. Supplementing the cleaning solutions with tauranol and poloxamer yielded a solution much more effective in stimulating bacterial detachment. Taurine an amino acid and an antioxidant protects corneal cells from osmotic stress [14]. Poloxamer 407 has an anti adhesive effect on gram negative and gram-positive bacteria thus inhibiting protein and debris from attaching to lens surface [15]. James E. Key also proposed positive results with a mixture of polyquarternium 10 and poloxamer [16]. Also ultrasonic lens cleaning devices have been reported and show a ray of hope in removing the adhered bacteria to the lens to a great extent. Hiti et al report positive results with microwave irradiation of *Acanthamoeba *species trophozoites from lens cases [17].

## Conclusion

Non adherence to lens cleaning protocol may invite microbial colonization of lens and its accessories leading to complications like keratitis which can cause permanent visual damage to the cornea. However, complications can be minimized if the subjects are instructed about the proper cleaning protocols and warned about the hazards of non-compliance. Besides, lens and its accessories be changed at regular intervals and in case of irritation, the use of lens be discontinued. The occurrence of contamination in the cleaning solutions cannot always be attributed to non-compliance but also raises a question on the percentage efficacy of the solutions in removing the protein debris from the lens. Therefore, modifications in the composition of the solutions are suggested at the manufacturers' level.

## Competing interests

The authors declare that they have no competing interests.

## Authors' contributions

All the authors read and approved the final manuscript. LT designed the study, performed the experimental work, conceived, drafted and edited the manuscript. GKB supervised the design, coordination of the study and helped to edit the manuscript. DKS helped in statistical analysis. TMM coordinated the study and helped to edit the manuscript.

## References

[B1] Kumar D (1985). A textbook on contact lens practice.

[B2] Stephen Foster C (2005). Smolin and Thoft's The Cornea.

[B3] Bharathi MJ, Ramakrishnan R, Meenakshi R, Mittal S, Shivkumar C, Srinivasan M (2006). Microbiological diagnosis of infective keratitis: comparative evaluation of direct microscopy and culture results. Br J Ophthalmol.

[B4] Willcox MDP, Harmis N, Cowell BA, Williams T, Holden BA (2001). Bacterial interactions with contact lenses; effects of lens material, lens wear and microbial physiology. Biomaterials.

[B5] Bruinsma GM, Rustema-Abbing M, de Vries J, Stegenga B, Mei HC Van der, Linden ML Van der, Hooymans JM, Busscher HJ (2002). Influence of wear and overwear on surface properties of etafilcon A contact lenses and adhesion of Pseudomonas aeruginosa. Invest Ophthalmol Vis Sci.

[B6] Collins MJ, Carney LG (1986). Patient compliance and its influence on contact lens wearing problems. Am J Optom Physiol Opt.

[B7] Yung AMS, Boost MV, Cho P, Yap M (2007). The effect of a compliance enhancement strategy (self-review) on the level of lens care compliance and contamination of contact lenses and lens care accessories. Clinical and Experimental Optometry.

[B8] Najjar DM, Aktan SG, Rapuano CJ, Laibson PR, Cohen EJ (2004). Contact lens-related corneal ulcers in compliant patients. American Journal of Ophthalmology.

[B9] Chun MW, Weissman BA (1987). Compliance in contact lens case. Am J Optom Physiol Opt.

[B10] Boost MV, Cho P (2005). Microbial flora of tears of orthokeratology patients, and microbial contamination of contact lenses and contact lens accessories. Optometry and Vision Science.

[B11] Steinemann TL, Fletcher M, Bonny AE, Harvey RA, Hamlin D, Zloty P, Besson M, Walter K, Gagnon M (2005). Over-the-Counter Decorative Contact Lenses: Cosmetic or Medical Devices? A Case Series. Eye & Contact Lens: Science & Clinical Practice.

[B12] Goodlaw E (1996). Risk of infection from sleeping with contact lens on: causes of risk. Optom Vis Sci.

[B13] Pinna A, Sechi LA, Zanetti S, Usai D, Delogu G, Cappurccinelli P, Carta F (2001). Bacillus cereus keratitis associated with contact lens wear. Ophthalmology.

[B14] Thimons JJ (2004). Examining taurine's role in ocular biochemistry and in contact lens wear". Contact Lens Spectrum.

[B15] Portoles M, Refojo MF, Leong FL (1994). Poloxamer 407 as a bacterial adhesive for hydrogel contact lenses. J Of Biomedical Materials Reasearch.

[B16] Key JE (2005). The lens care solution to patient retention. Review of Ophthalmology.

[B17] Hiti K, Walochnik J, Faschinger C, Schober EMH, Aspock H (2001). Microwave treatment of contact lens cases contaminated with Acanthamoeba. Cornea.

